# Ginsenoside Rb2 Alleviates Hepatic Lipid Accumulation by Restoring Autophagy via Induction of Sirt1 and Activation of AMPK

**DOI:** 10.3390/ijms18051063

**Published:** 2017-05-19

**Authors:** Qi Huang, Ting Wang, Liu Yang, He-Yao Wang

**Affiliations:** 1 State Key Laboratory of Drug Research, Shanghai Institute of Materia Medica, Chinese Academy of Sciences, Shanghai 201203, China; 2 University of Chinese Academy of Sciences, Beijing 100049, China

**Keywords:** ginsenoside Rb2, NAFLD, type 2 diabetes, hepatosteatosis, autophagy, AMPK, sirt1

## Abstract

Although Panax ginseng is a famous traditional Chinese medicine and has been widely used to treat a variety of metabolic diseases including hyperglycemia, hyperlipidemia, and hepatosteatosis, the effective mediators and molecular mechanisms remain largely unknown. In this study we found that ginsenoside Rb2, one of the major ginsenosides in Panax ginseng, was able to prevent hepatic lipid accumulation through autophagy induction both in vivo and in vitro. Treatment of male db/db mice with Rb2 significantly improved glucose tolerance, decreased hepatic lipid accumulation, and restored hepatic autophagy. In vitro, Rb2 (50 µmol/L) obviously increased autophagic flux in HepG2 cells and primary mouse hepatocytes, and consequently reduced the lipid accumulation induced by oleic acid in combination with high glucose. Western blotting analysis showed that Rb2 partly reversed the high fatty acid in combination with high glucose (OA)-induced repression of autophagic pathways including AMP-activated protein kinase (AMPK) and silent information regulator 1 (sirt1). Furthermore, pharmacological inhibition of the sirt1 or AMPK pathways attenuated these beneficial effects of Rb2 on hepatic autophagy and lipid accumulation. Taken together, these results suggested that Rb2 alleviated hepatic lipid accumulation by restoring autophagy via the induction of sirt1 and activation of AMPK, and resulted in improved nonalcoholic fatty liver disease (NAFLD) and glucose tolerance.

## 1. Introduction

Panax ginseng is one of the most commonly used traditional herbal tonics [1,2]. Ginsenosides are the major active component of ginseng and are known to responsible for various pharmacological effects of Panax ginseng, such as anti-inflammation, anti-tumor, and nonalcoholic fatty liver disease (NAFLD) prevention [3,4,5]. Rb2 is considered to be the most abundant ginsenoside in Panax ginseng and has been reported to improve hyperlipidemia in streptozotocin-diabetic rats [6]. In addition, Rb2 has been proven to be an AMP-activated protein kinase (AMPK) activator and able to inhibit palmitate-induced gluconeogenesis in H4IIE liver cells [7]. Recently, researchers found that Rb2 could alleviate ethanol-induced steatosis by upregulating the expression of silent information regulator 1 (sirt1) and improving mitochondrial function by increasing the activity of sirt1 in hepatocytes [8,9]. The liver is an important organ for the production and clearance of lipids. Hepatic lipid overload can result in the occurrence and development of NAFLD. NAFLD now affects more than 20% of the general population and is an emerging risk factor for the development of diabetes, liver fibrosis, and cardiovascular disease. Although drug therapies with sufficient efficacy and safety for NAFLD have not been fully established, it is clear that lipid homeostasis is critical for the prevention and treatment of NAFLD [10].

Autophagy is important for regulating organelle function and insulin signaling and defective autophagy is closely related to insulin resistance in obesity [11]. Recent studies suggest that impaired hepatic autophagic activity in the liver increase lipid accumulation and eventually results in NAFLD [12,13], while activating autophagy promotes lipid clearance and exerts a hepatoprotective effect [14]. Hepatic autophagy is regulated mainly through the AMP-activated protein kinase (AMPK)-mechanistic target of rapamycin (mTOR) and silent information regulator 1 (sirt1)-Forkhead box O (FOXO) pathways, which are activated by the increment of the adenosine diphosphate (ADP)/adenosine triphosphate (ATP) ratio and nicotinamide adenine dinucleotide (NAD^+^), respectively [15,16]. Several studies suggest that the activation of AMPK or induction of sirt1 could restore the impaired autophagy and alleviate the hepatic lipid accumulation [17,18,19].

In this study we aimed to identify the beneficial effects of Rb2 on NAFLD and tested the hypothesis that Rb2 promoted hepatic autophagy via the AMPK/Sirt1-dependent pathways and consequently alleviated hepatic lipid accumulation.

## 2. Results

### 2.1. Rb2 Alleviated Hepatic Steatosis and Improved Glucose Tolerance in db/db Mice

**Figure 1:**
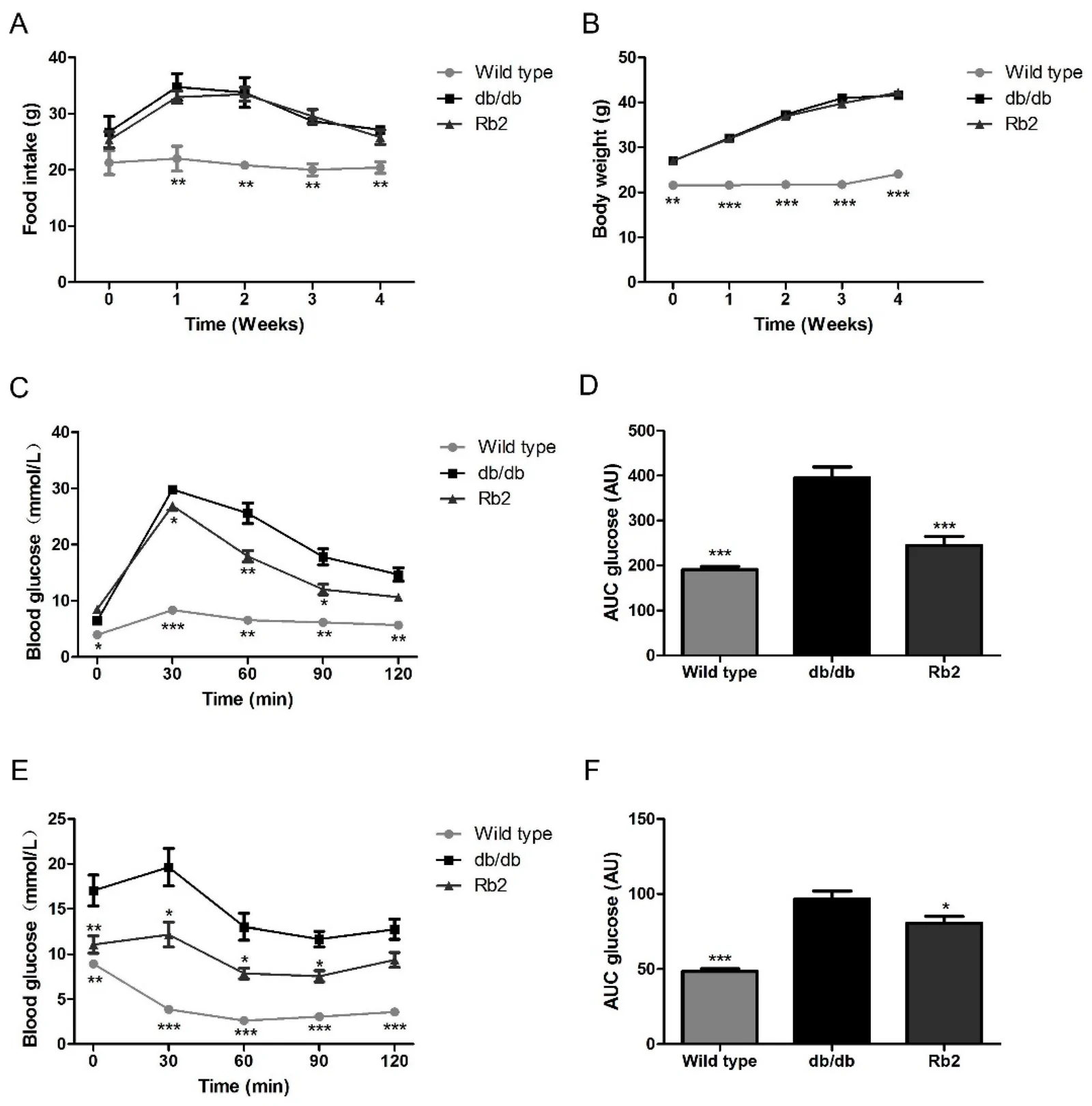
Metabolic effects of Rb2 on db/db mice. The alteration of food intake (**A**) and body weight (**B**) in 6-week-old db/db mice and wild type mice were monitored during the 4-week administration. db/db mice in two different groups were treated with either Rb2 (10 mg/kg) or saline. At day 21, the glucose tolerance test was performed after an oral administration of glucose (1 g/kg body weight, **C**,**D**). At day 24, the insulin tolerance test was performed after intraperitoneal injection of insulin (0.8 IU/kg body weight, **E**,**F**). The tail vein blood was collected and glucose concentrations were measured at 0, 30, 60, 90, and 120 min. Wild type: Wild type mice as normal control, db/db: Vehicle-treated db/db mice, Rb2: Rb2 (10 mg/kg body weight)-treated db/db mice. Data are expressed as mean ± standard error (SE) from 10 animals per group. * *p* < 0.05, ** *p* < 0.01, and *** *p* < 0.001 compared with vehicle-treated db/db mice group. AU, any unit. AUC, area under the curve, the basal glucose values of each mouse in oral administration of glucose (OGTT) and intraperitoneal insulin tolerance test (IPITT) AUC is set to 1.

The db/db mouse is a recognized animal model with over-weight, severe glucose intolerance, and fatty liver due to the leptin receptor-deficiency [20]. Food intake and body weight were not significantly affected during the 4-week administration of Rb2 in db/db mice (Figure 1A,B). Since type 2 diabetes (T2DM) is characterized with impaired glucose intolerance and insulin resistance, the oral administration of glucose (OGTT) and intraperitoneal insulin tolerance test (IPITT) were employed to roughly assess the beneficial metabolic phenotype of Rb2. Results showed that Rb2 administration significantly improved glucose tolerance and insulin sensitivity in db/db mice (Figure 1C–F). As shown in Table 1, the markedly increased levels of triglycerol (TG), total cholesterol (TCH), and glucose in the serum of db/db mice were partly reversed by Rb2. In addition, 4-week Rb2 administration did not alter the weight of epididymal fat and perirenal fat, indicating Rb2 had little effect on fat loss.

**Figure 2:**
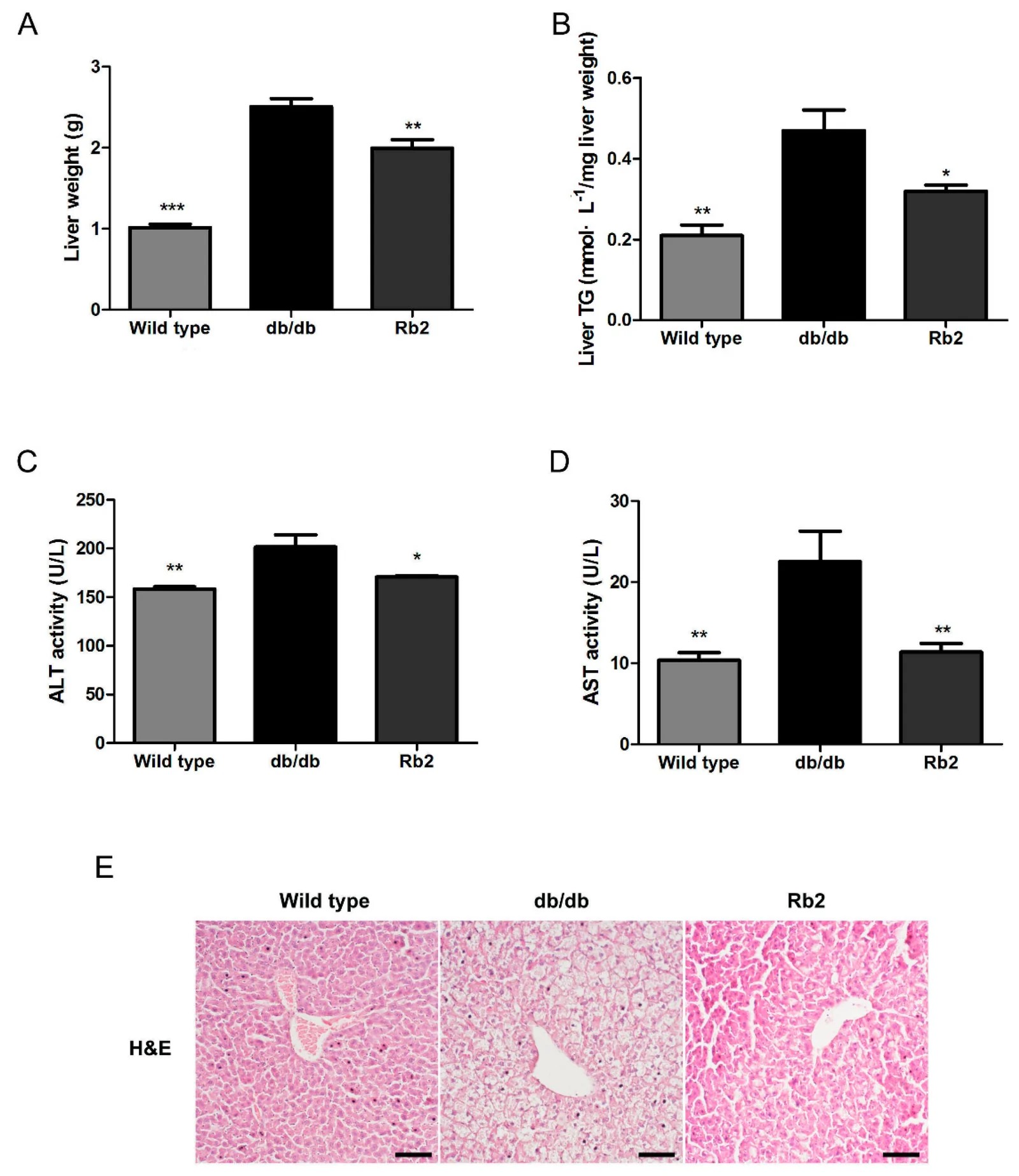
Rb2 attenuated hepatic steatosis in db/db mice. At the end of the 4-week administration, the effect of Rb2 on liver weight (**A**) and triglycerol (TG)content of the liver (**B**) in wild type and db/db mice were assessed. Serum levels of alanine aminotransferase (ALT, **C**) and aspartate aminotransferase (AST, **D**) were calculated. Liver sections were stained with hematoxylin and eosin (H&E), and representative images around the perisinusoidal spaces were captured (Scale bars = 100 µm) (**E**). Wild type: Wild type mice as normal control, db/db: Vehicle-treated db/db mice, Rb2: Rb2 (10 mg/kg body weight)-treated db/db mice. Data are expressed as mean ± SE from 10 animals per group. * *p* < 0.05 and ** *p* < 0.01 compared with db/db group.

Since liver plays a key role in energy homeostasis, and obesity-induced NAFLD impairs the function of the liver [21], we further evaluated whether Rb2 could improve the liver function of db/db mice. Weight and TG content of the liver in db/db mice were obviously at higher levels than wild type mice, whereas Rb2 significantly ameliorated the fatty liver phenotype (Figure 2A,B). In accord with these results, Rb2-treated mice showed improved liver function than db/db mice as the serum activity of alanine aminotransferase (ALT) and aspartate aminotransferase (AST) were significantly decreased (Figure 2C,D). Moreover, less lipid particle accumulation and improved macrovesicular steatosis in Rb2-administrated mice were observed with hematoxylin and eosin (H&E) staining of liver sections (Figure 2E).

### 2.2. Rb2 Increased Expression of Sirt1 and Phosphorylation of AMP-Activated Protein Kinases (AMPK) and Restored the Impaired Hepatic Autophagy in db/db Mice

**Figure 3:**
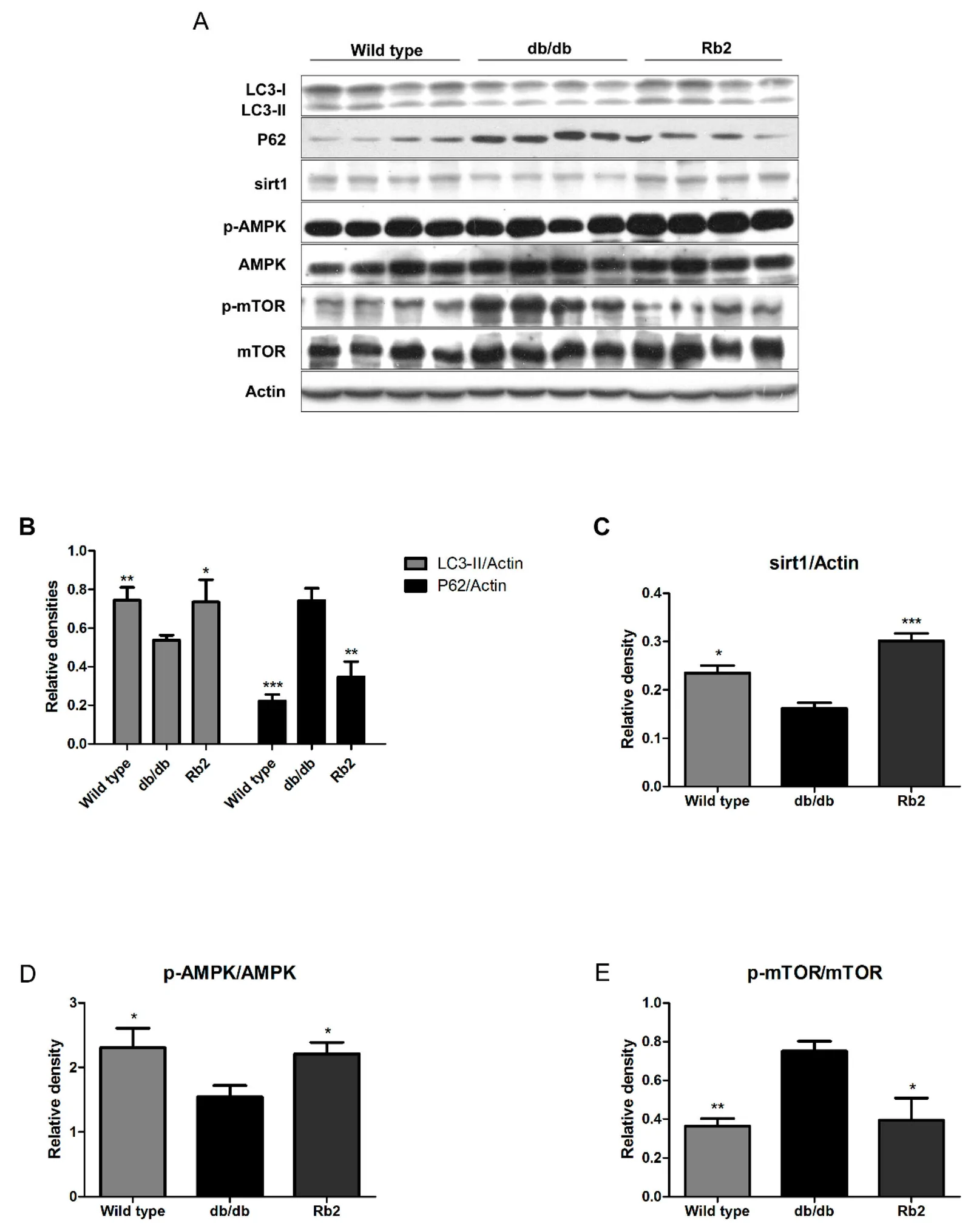
Effect of Rb2 on the hepatic autophagy pathway. Autophagy marker proteins from the liver were evaluated by Western blotting analysis (**A**). Bar chart showing the semi-quantitative integrated optical density (arbitrary units) of microtubule-associated protein 1 light chain 3II (LC3-II) and polyubiquitin-binding protein p62 (P62) normalized by actin (**B**), silent information regulator 1 (sirt1) normalized by actin (**C**), phosphorylated AMP-activated protein kinase (p-AMPK) normalized byAMPK (**D**), phosphorylated mammalian target of rapamycin (p-mTOR) normalized by mTOR (**E**). Wild type: Wild type mice as normal control, db/db: Vehicle-treated db/db mice, Rb2: Rb2 (10 mg/kg body weight)-treated db/db mice. Data are expressed as mean ± SE from three independent experiments. * *p* < 0.05, ** *p* < 0.01, and *** *p* < 0.001 compared with db/db group.

Pronounced or chronically sustained lipid challenges suppress both AMPK- and sirt1-mediated autophagic pathways in hepatocytes, leading to decreased autophagic activity and consequent accumulation of intracellular lipids [18,22]. The autophagic pathways in liver tissues were evaluated by Western blot analysis (Figure 3A). Integrated optical density analysis showed that the level of microtubule-associated protein 1 light chain 3II (LC3-II) was dramatically reduced, while P62 was significantly higher in vehicle-treated db/db mice than that in the wild type group. Additionally, the levels of sirt1 and phosphorilated AMPK (p-AMPK)/AMPK ratio decreased, while mTOR was activated in the liver of vehicle-treated db/db mice, suggesting that the autophagic activity obviously decreased in the liver of db/db mice. Rb2 treatment significantly upregulated the level of LC3-II and decreased the level of P62 (Figure 3B). Furthermore, sirt1 expression (Figure 3C) and the p-AMPK/AMPK ratio (Figure 3D) were elevated in Rb2-treated db/db mice, whereas the phosphorilated mTOR (p-mTOR)/mTOR ratio (Figure 3E) was decreased compared with the vehicle-treated db/db mice. The results indicated that long-term Rb2 administration may promote hepatic autophagic activity through both the sirt1 and AMPK pathways.

### 2.3. Rb2 Dose- and Time-Dependently Promoted Autophagic Flux in Hepatic Cells

**Figure 4:**
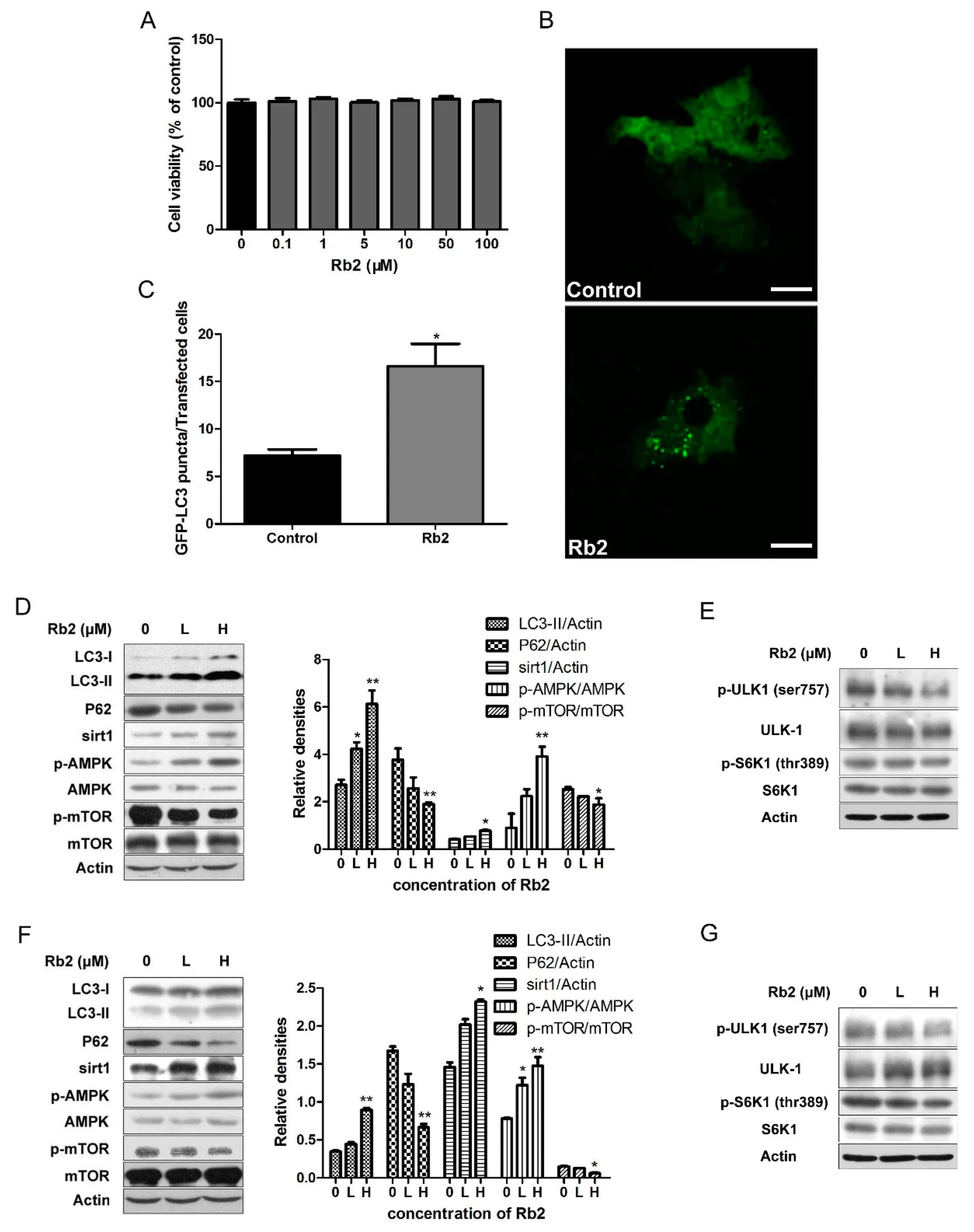
Rb2 dose-dependently stimulated autophagy pathways in hepatocytes. Effect of Rb2 (1, 0.1, 1, 10, 50, and 100 µmol/L) on HepG2 cell (*n* = 6) viability (**A**) was determined by 5-diphenyltetrazoliumbromide (MTT). The effect of Rb2 (50 µmol/L) on green fluorescent protein (GFP)-tagged LC3 punctas formation in HepG2 cells was monitored by fluorescence confocal microscopy (**B**, Scale bars = 5 µm), and the amount of GFP-LC3 punctas per transfected cell was quantified (**C**). The levels of autophagy marker and regulatory proteins were evaluated by Western blotting analysis after Rb2 (0: Dimethylsulphoxide (DMSO), L: 10 µmol/L, H: 50 µmol/L) treatment for 12 h in HepG2 cells (**D**,**E**) and primary mouse hepatocytes (**F**,**G**). Data are expressed as mean ± SE from three independent experiments. * *p* < 0.05 and ** *p* < 0.01 compared with vehicle (DMSO)-treated group (Control).

**Figure 5:**
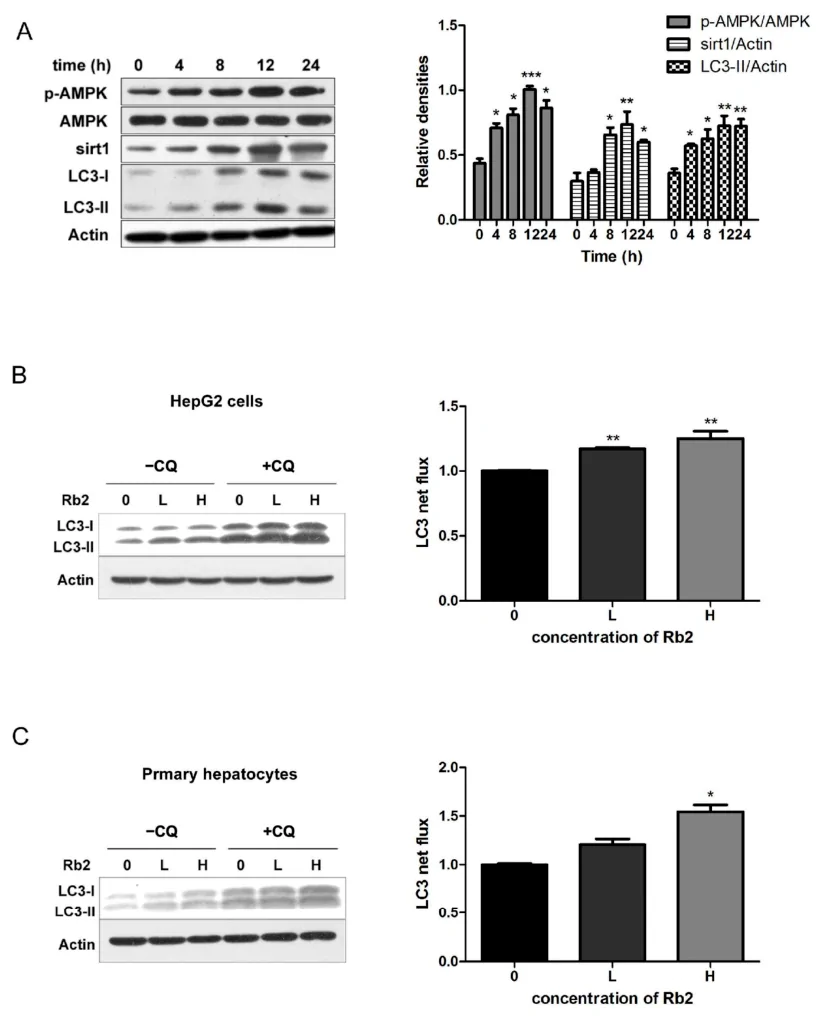
Rb2 promoted autophagic flux by increasing sirt1 expression and AMPK phosphorylation in a time- and dose-dependent manner in hepatocytes. The levels of LC3-II, P62, sirt1, and the p-AMPK/AMPK ratio were evaluated by Western blotting analysis after Rb2 (50 µmol/L) treatment for 0, 4, 8, 12, and 24 h in HepG2 cells (**A**). Autophagic flux was evaluated by Western blotting analysis after Rb2 (0: DMSO, L: 10 µmol/L, H: 50 µmol/L) treatment with and without chloroquine (CQ) (25 µmol/L) for 12 h in HepG2 cells (**B**) and primary mouse hepatocytes (**C**). Data are expressed as mean ± SE from three independent experiments. * *p* < 0.05, ** *p* < 0.01, and *** *p* < 0.001 compared with vehicle (DMSO)-treated group (Control).

The 5-diphenyltetrazoliumbromide (MTT) results showed that Rb2 treatment at a concentration up to 100 µmol/L had no cytotoxic effects on HepG2 cells (Figure 4A). To investigate the effect of Rb2 on hepatic autophagy flux in vitro, HepG2 cells transiently expressing green fluorescent protein (GFP)-tagged LC3 were treated with Rb2. A marked increase of GFP-LC3 punctas per transfected cell in Rb2-treated cells was observed (Figure 4B,C). Furthermore, Rb2 effectively upregulated the levels of LC3-II, and suppressed intracellular accumulation of P62 in a dose-dependent manner in HepG2 cells. We then analyzed the expression levels of Sirt1, p-AMPK, and p-mTOR, the upstream regulator of the autophagic pathway. The treatment of HepG2 cells with Rb2 resulted in an obvious increase of sirt1 expression and the p-AMPK/AMPK ratio, while the p-mTOR/mTOR ratio was down-regulated dose-dependently. (Figure 4D). Ribosomal protein S6 kinase 1 (S6K1) is an important downstream effector of the mTOR complex. It is also shown that the inhibitory phosphorylation of UNC-5 like autophagy activating kinase 1 (ULK1) at ser757 by the mTOR complex is decreased upon the autophagy-inducing condition in an AMPK-dependent manner [23]. To further confirm the role of AMPK-mTOR in this autophagy-indcing effect, we assessed the levels of phosphorylated S6K1 (thr389) and ULK1 (ser757) under the action of Rb2. The results showed that both phosphorylated S6K1 (thr389) and phosphorylated ULK1 (ser757) decreased after 12 h treatment of Rb2 in HepG2 cells. The similar pattern was also observed in mouse primary hepatocytes (Figure 4F,G). In addition, sirt1 expression and the p-AMPK/AMPK ratio in HepG2 cells increased time-dependently and started early at 4 h followed by Rb2 incubation (Figure 5A).

To assess the autophagy flux, we pharmacologically blocked autophagosome-lysosome fusion using Chloroquine diphosphate (CQ) (25 µmol/L), an inhibitor of lysosomal function. Increased autophagic flux was observed upon exposure to Rb2 (10, 50 µmol/L), either in the presence or absence of 25 µM CQ in both HepG2 cells and primary hepatocytes (Figure 5B,C). The results confirmed the real effects of Rb2 on autophagic flux restoration in our experiments.

### 2.4. The Inhibitory Effect of Rb2 on Lipid Accumulation Depends on a Coordinate Increase in Hepatic Autophagy

**Figure 6:**
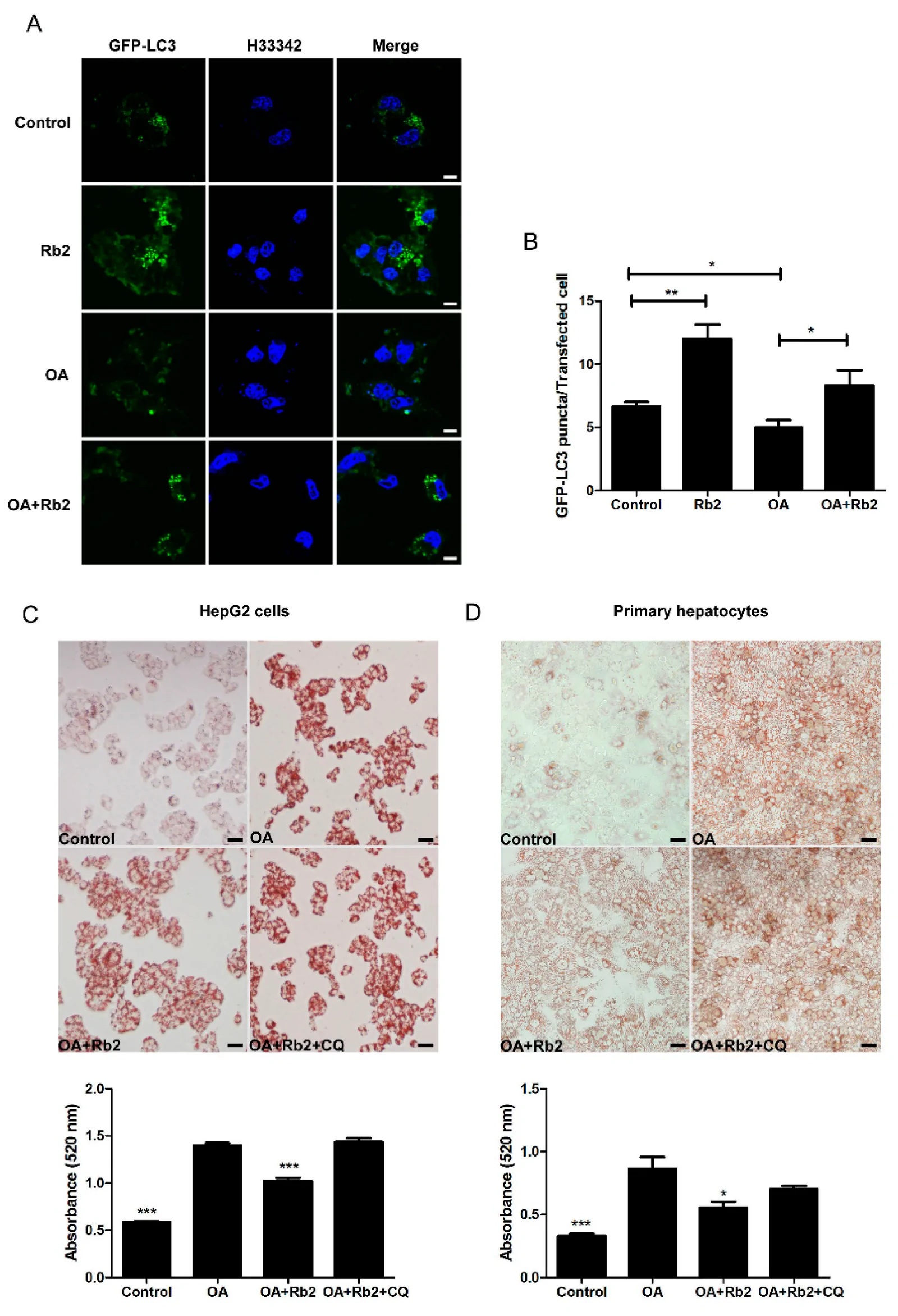
Rb2 suppressed high fatty acid in combination with high glucose (OA-induced hepatosteatosis via the upregulation of autophagy. The effect of OA with and without Rb2 on GFP-LC3 punctas formation in HepG2 cells were monitored by fluorescence confocal microscopy (**A**, Scale bars = 5 µm), and the amount of GFP-LC3 punctas per transfected cell was quantified (**B**). * *p* < 0.05 and ** *p* < 0.01 compared with indicated two groups. HepG2 cells (**C**) and primary mouse hepatocytes (**D**) were pretreated with 50 µmol/L Rb2, with or without CQ (25 µmol/L) for 4 h before OA (1 mmol/L for HepG2 and 2 mmol/L for primary mouse hepatocytes) exposure for 12 h, and lipid accumulation were visualized (Scale bars = 20 µm) and quantified by oil red O (ORO) staining. Data are expressed as mean ± SE from three independent experiments. * *p* < 0.05, ** *p* < 0.01, and *** *p* < 0.001 compared with the group that was pretreated with vehicle (Control) prior to OA exposure.

To determine whether autophagy induced by Rb2 is directly involved in reducing the intracellular lipid content, hepatocytes treated with oleic acid (1 mmol/L for HepG2 and 2 mmol/L for primary hepatocytes) in combination with 33.3 mmol/L glucose (OA) for 12 h as an in vitro model with over-accumulated hepatic lipids was employed. The nucleus and the autophagosomes were labeled with Hoechst33342 (H33342) and GFP-LC3 punctas, respectively. The number of GFP-LC3 punctas significantly decreased in OA-treated HepG2 cells while Rb2 partly reversed this autophagic activity decline induced by sustained lipid challenges (Figure 6A,B). Next, lipid droplets in HepG2 cells (Figure 6C) and primary hepatocytes (Figure 6D) were visualized and quantified by Oil red O (ORO) staining. Exposure to high levels of fatty acid and glucose dramatically increased intracellular lipid accumulation in HepG2 cells and primary hepatocytes. However, pretreatment with Rb2 for 4 h obviously attenuated OA-induced lipid accumulation in both hepatocytes. In addition, co-treatment with CQ (25 µmol/L) abolished the reduction effect of Rb2 on lipid accumulation, indicating that the capacity of Rb2 to alleviate hepatic lipid accumulation is tightly associated with the promotion of autophagy. The findings suggested that Rb2 treatment could help to prevent hepatic lipid accumulation through promoting autophagy.

### 2.5. Rb2 Restored Impaired Hepatic Autophagy through Increasing Expression of Sirt1 and Phosphorylation of AMPK in Cultured Steatotic Hepatocytes

**Figure 7:**
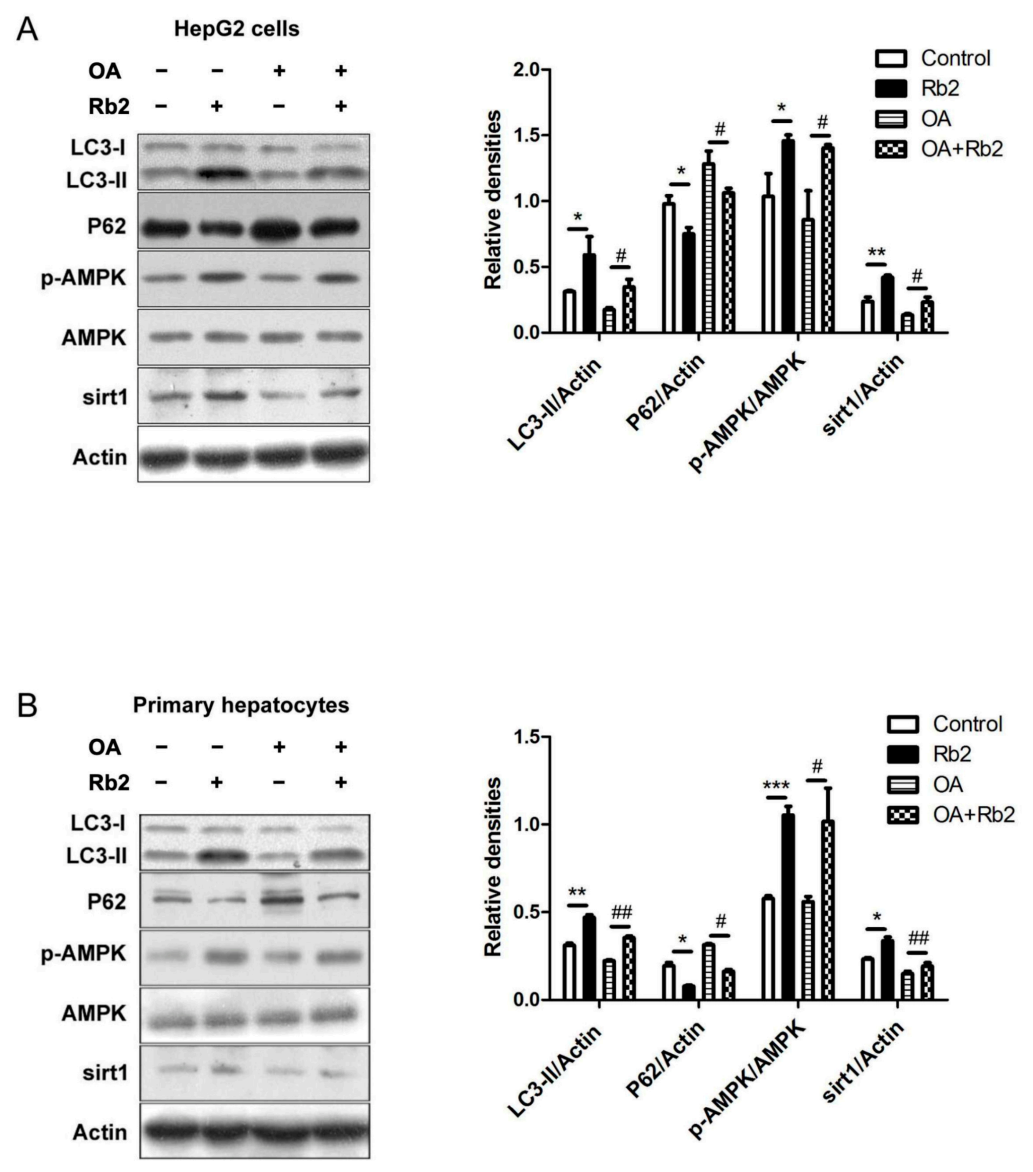
The effect of Rb2 on OA-induced hepatic autophagy dysfunction through the upregulation of sirt1 expression and AMPK phosphorylation. HepG2 cells (**A**) and primary mouse hepatocytes (**B**) were pretreated with 50 µmol/L Rb2 for 4 h before OA (33.3 mmol/L glucose in combination with 1 mmol/L OA for HepG2 and 2 mmol/L OA for primary hepatocytes) exposure for 12 h, and levels of LC3-II, P62, sirt1, p-AMPK, and AMPK were detected by Western blotting analysis. Data are expressed as mean ± SE from three independent experiments. * *p* < 0.05, ** *p* < 0.01, and *** *p* < 0.001 compared with vehicle-treated group (Control). ^#^ *p* < 0.05 and ^##^ *p* < 0.01 compared with OA-treated group (Control).

Sirt1 induces autophagy through deacetylating autophagy mediators such as LC3 and Autophagy-related gene 5(ATG5), and phosphorylated AMPK promotes autophagy via the inhibition of mTOR. To further test our hypothesis that sirt1 and AMPK act as moderators for the effect of Rb2 on autophagy induction, we determined the levels of sirt1 and the p-AMPK/AMPK ratio, as well as the relevant levels of LC3-II and P62 in OA-treated HepG2 cells (Figure 7A) and primary hepatocytes (Figure 7B) in the presence or absence of Rb2. A significant decrease of the sirt1 protein level was observed in OA-treated hepatocytes, while Rb2 treatment attenuated this decline. The ratio of p-AMPK/AMPK was upregulated by Rb2 either in the presence or in the absence of OA. Meanwhile, OA-induced downregulation of LC3-II was restored, while the increased level of P62 was adjusted close to normalcy in Rb2-treated cells.

**Figure 8:**
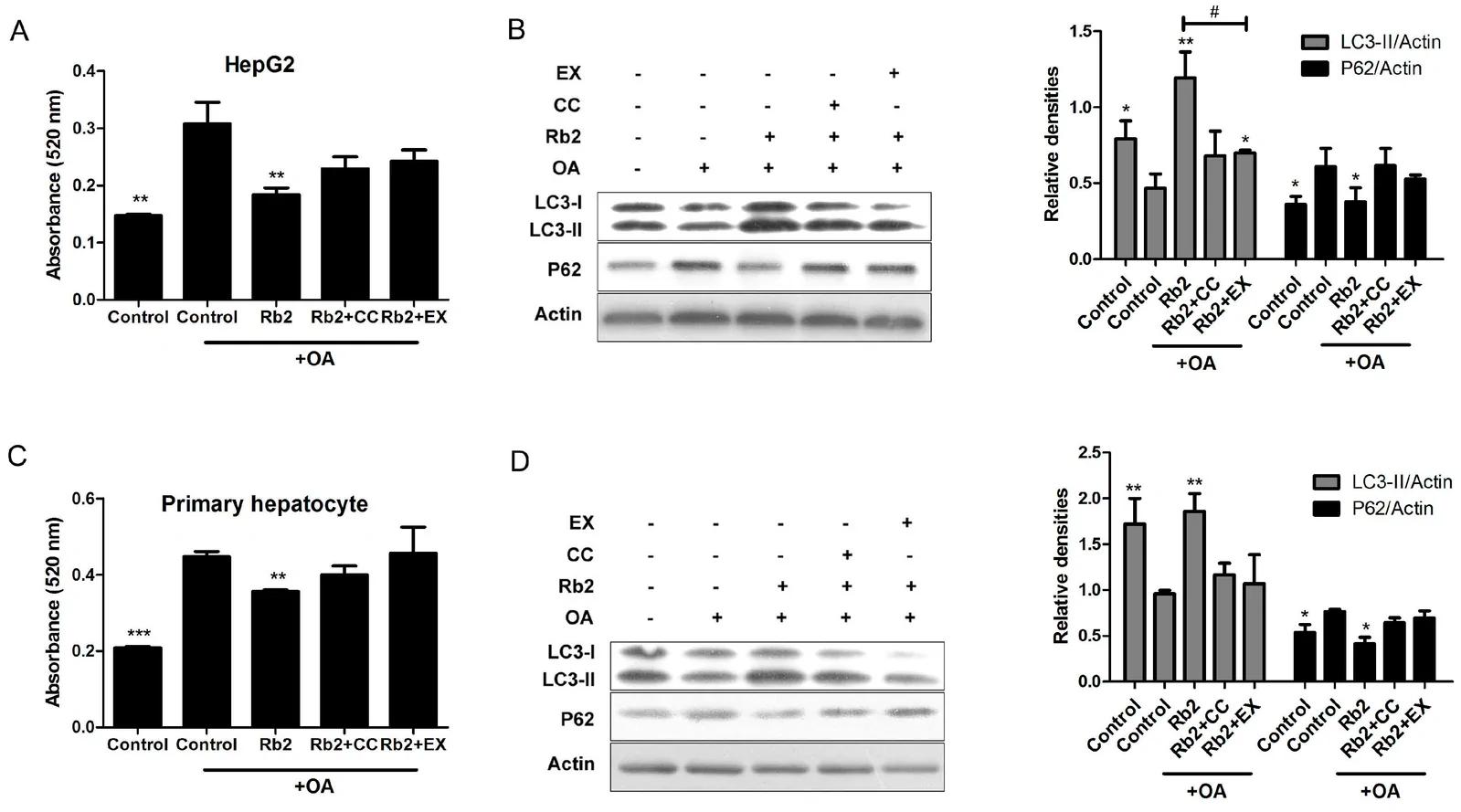
Inhibition of sirt1 and AMPK blocked Rb2-induced hepatic autophagy. HepG2 cells (**A**,**B**) and primary mouse hepatocytes (**C**,**D**) were pretreated with 50 µmol/L Rb2 for 4 h in the presence or absence of the sirt1 inhibitor EX-528 (EX) and the specific AMPK inhibitor Compound C (CC), and then subjected to OA (1 mmol/L for HepG2 and 2 mmol/L for primary mouse hepatocytes) exposure for 12 h. For lipid content determination, intracellular TG were stained by Oil red O (ORO). ORO was then eluted with isopropanol and the optical absorbance of the eluate was measured at 520 nm (*n* = 3). Data are expressed as mean ± SE from three independent experiments. * *p* < 0.05, ** *p* < 0.01 and *** *p* < 0.001 compared with the group that was pretreated with vehicle (DMSO) prior to OA exposure. ^#^ *p* < 0.05 compared between the two indicated groups. For autophagic activity evaluation, levels of LC3-II and P62 were detected and quantified by Western blotting analysis.

Furthermore, quantified ORO staining showed that theimproving effect of Rb2 on lipid accumulation and autophagic markers (LC3-II and P62) were partly blocked by either the specific sirt1 inhibitor EX-528 (EX) or the specific AMPK inhibitor Compound C (CC) in HepG2 cells (Figure 8A,B) and primary mouse hepatocytes (Figure 8C,D). We therefore concluded that Rb2 restored autophagy and attenuated lipid accumulation mostly through AMPK activation and sirt1 induction in cultured steatotic hepatocytes.

## 3. Discussion

Panax ginseng is one of the most famous medical herbs in oriental countries. Although Panax ginseng is known for its beneficial effects on fatty liver and T2DM [24,25,26], the effective mediators and molecular mechanisms remain largely unclear. Studies have shown that ginsenoside Rb2 exerted improvement effects on hyperlipemia and ethanol-induced steatosis [7,8]. In this study, we examined the protective effect of Rb2 on NAFLD and insulin sensitivity in db/db mice, a murine model frequently used for NAFLD and T2DM. Our results showed that a 4-week course of Rb2 administration significantly reduced hepatic steatosis and improved metabolic homeostasis in db/db mice. Notably, those effects were not accompanied by food intake decrease or body weight change. In addition, increased protein levels of sirt1 and p-AMPK, along with improvedhepatic autophagy were observed in db/db mice treated with Rb2. The underlying mechanisms of NAFLD are complex [27,28]. Hepatic lipid over-accumulation caused by the imbalance between lipid availability and lipid disposal is considered to be an important mechanism of NAFLD [29]. Currently, emerging evidence suggests that the progression of NAFLD is associated with impaired hepatic autophagic activity in both ob/ob [11] and high fat diet (HFD)-fed mice [30]. Autophagy is a self-renewal pathway that mediates the degradation of cytoplasmic contents in lysosomes, thus keeping cellular metabolism homeostasis [31,32]. Particularly, the autophagy process degrading lipid droplets is termed lipophagy. The induction of hepatic lipophagy through the sirt1/FOXO1 or AMPK/mTOR pathways helps to facilitate lipid droplet clearance and prevent fatty liver disease [22,23,33]. Our results suggested that the impaired hepatic autophagy may be tightly associated with NAFLD, as Rb2 significantly improved impaired hepatic autophagy and attenuated the hepatic steatosis in db/db mice.

Defective autophagy in the liver may contribute to hepatic lipid accumulation [34], which in turn has the ability to damage autophagic function [11]. In our in vitro experiments, several important lines of evidence have been provided to demonstrate that Rb2 induces autophagic activity and is directly involved in reducing hepatic lipid content in cultured hepatic cells. Firstly, Rb2 dose- and time-dependently promoted autophagic flux in both HepG2 cells and mouse primary hepatocytes. Secondly, Rb2 obviously reduced the intracellular lipid accumulation and restored the impaired autophagy in cultured steatotic hepatocytes, which represents both lipid accumulation and autophagy dysfunction. Importantly, this improvement effect was abolished by a lysosomal function inhibitor (CQ), which demonstrated that Rb2 was a positive regulator of the upstream pathway of lysosomes in macroautophagy. Thirdly, the reduction effect of Rb2 on hepatic lipid accumulation relied on the sirt1 and AMPK pathways, which were considered to be two critical regulatory pathways of autophagy. The expression of sirt1 and the phosphorylation of AMPK were upregulated by Rb2, and this stimulation effect was blocked by either the sirt1 specific inhibitor EX-527 or AMPK specific inhibitor compound C. Together the results demonstrated that Rb2 induced sirt1 expression and activated AMPK to enhance autophagy promotion and led to the decrease of hepatocellular lipid droplets in steatotic hepatocytes.

While the effect of Rb2 on autophagy has not been reported before, some other active ginsenosides were considered to be autophagy regulators with various benefits. For instance, Rh2 was shown to induce autophagy in hepatocellular carcinoma and exert an anti-tumor effect [35]. Most recently, Rg2 was reported to exhibit neuronal and metabolic benefits through autophagy induction [36]. Interestingly, in a report it was shown that Rg1 inhibited angiotensin II-induced podocyte autophagy via the AMPK/mTOR/PI3K pathway [37]. However in another study, Rg1 was demonstrated to reduce aldosterone-induced autophagy via the AMPK/mTOR pathway in NRK-52E cells [38]. Similarly, Rb1 was reported to have positive [39] and negative [40] effects on neuronal autophagy, respectively, in two different papers. Although the difference in cell types and observation indexes may contribute to these inconsistent results, further work is needed to identify the specific effects of gisenosides on autophagy and the underlying mechanisms.

## 4. Materials and Methods

### 4.1. Reagents

Ginsenoside Rb2 (purity >98.0%) was purchased from Shanghai Tauto Biotech Co., Ltd (Shanghai, China). Dulbecco’s Modified Eagle’s medium (DMEM), William’s E Medium, and Fetal Bovine Serum (FBS) for cell culture were obtained from Thermo Fisher Scientific (San José, CA, USA). MTT, bovine insulin, ORO powder, collagenase IV, and dexamethasone were obtained from Sigma-Aldrich (Saint Louis, MO, USA). Compound C, EX-527, and CQ were obtained from Medchem Express (Shanghai, China). Primary antibodies (anti-LC3, anti-P62, anti-sirt1, anti-AMPK, anti-p-AMPK, anti-mTOR, anti-p-mTOR) were from Cell Signaling Technology (Beverly, MA, USA). Primary antibody anti-actin was obtained from Sigma-Aldrich. Secondary antibodies anti-mouse IgG and anti-rabbit IgG were obtained from Jackson Laboratory (Bar Harbor, ME, USA). The plasmid expressing GFP-LC3 was a kind gift from Professor Xin Xie (Shanghai institute of materia medica, Shanghai, China).

### 4.2. Animals

C57BL/KsJ-Lepdb (db/db) mice and their lean littermates (wild type) were obtained from Jackson Laboratory. Six-week-old male mice were maintained at 22 ± 2 °C with 60 ± 5% relative humidity, and under a 12 h light/dark cycle with free access to water and regular chow diet. The db/db mice (*n* = 10/group) were daily administrated (intraperitoneal injection) with Rb2 (10 mg/kg) or saline (vehicle). Meanwhile, the wild type was treated with vehicle in an identical manner as the normal control. Body weight and food intake were monitored twice a week over a 4-week course of treatment. All of the animal experiments were approved by the Institutional Animal Care and Use Committee in Shanghai Institute of Materia Medica (No: SIMM-2016-04WHY-14).

### 4.3. Metabolic and Biochemical Analysis

Mice were fasted for 14 h to perform OGTT at day 21 and for 6 h to perform IPITT at day 24 as previously described [41]. The tail vein blood was collected and glucose concentrations were measured using Accu-Chek (Roche, Basel, Switzerland). At the end of the experiment, mice were fasted for 4 h and blood samples were collected from the orbital venous plexus before the mice were sacrificed. Livers were removed and kept in 4% paraformaldehyde or liquid nitrogen. Serum levels of glucose, alanine aminotransferase (ALT), alanine aminotransferase (AST), triglyceride (TG), and total cholesterol (TCH) were quantified using commercial kits (Mingdian, Nangjing, China). Serum insulin levels were determined using an enzyme-linked-immunosorbent assay (Millipore, Billerica, MA, USA).

### 4.4. Cell Culture

HepG2 cells were purchased from the American Type Culture Collection and maintained in DMEM supplemented with 10% FBS in a humidified incubator (5% CO_2_) at 37 °C. Primary mouse hepatocytes were isolated from eight-week-old male C57BL mice according to the 2-step collagenase IV perfusion method and maintained in William’s E Medium complete medium as previously reported [42]. To create an in vitro model that is accompanied with dysfunctional autophagic pathways and lipid accumulation, hepatocytes were exposed to OA (1 mmol/L for HepG2 cells and 2 mmol/L for primary hepatocytes) in combination with 33.3 mmol/L glucose for 12 h [22].

### 4.5. 5-Diphenyltetrazoliumbromide (MTT) Assay

HepG2 cells (2 × 10^4^ cells/well) were plated onto 96-well plates and cultured overnight in growth medium. The cells were then incubated with the indicated concentration of Rb2 for 48 h before the MTT reagent (0.5 mg/mL) was added to the media and incubated for another 4 h. The media was then removed and the formed formazan crystal was dissolved with 100 µL dimethylsulphoxide (DMSO). The absorbency at a wavelength of 492 nm was measured using a FlexStation 3 microplate reader (Molecular Devices, Sunnyvale, CA, USA).

### 4.6. Western Blot Analysis

HepG2 cells, primary hepatocytes, and mouse liver tissues were lysed in RIPA Lysis Buffer (Beyotime, Jiangsu, China). The protein concentrations of the lysates were determined with the BCA Protein Assay Kit (Beyotime). Equal amounts of total protein samples were subjected with sodium dodecyl sulphate-polyacrylamide gel electrophoresis (SDS-PAGE) and electroblotted onto polyvinylidene fluoride membranes (PVDF, Millipore). Membranes were subsequently probed as indicated with primary antibodies (diluted 1:1000) overnight at 4 °C. After incubation with horseradish-peroxidase-conjugated secondary antibody (diluted 1:10,000) for 2 h at room temperature, the blots were then visualized using chemiluminescence (ECL) detection reagents (Thermo Fisher Scientific). Data were analyzed by integrated optical density.

### 4.7. Detection of Autophagic Flux

Autophagic flux represents the dynamic process of autophagy [43]. For fluorescence visualization, the plasmid driving the expression of GFP-LC3 was transfected into HepG2 cells with X-tremeGENE HP DNA Transfection Reagent (Roche Diagnostic Systems, Branchburg, NJ, USA) for 12 h. Cells were then subject to treatment of OA in combination with high glucose for 12 h and fixed with 4% paraformaldehyde prior to labeling the nucleus with Hoechst 33342 (Sigma, St. Louis, MO, USA). Images were acquired on a FV10 confocal microscope (Olympus, Tokyo, Japan). The numbers of GFP-LC3 punctas representing autophagosomes were counted. For protein quantification, the level of LC3-II was determined by Western blot analysis. Autophagy flux was defined as the difference in the amount of LC3-II in the absence and presence of CQ (25 µmol/L).

### 4.8. Oil Red O (ORO) Staining and Quantification of Hepatic TG Contents

HepG2 cells and primary mouse hepatocytes were fixed with 4% paraformaldehyde and stained in ORO solution (10 mg ORO powder dissolved in 1 milliliter of 60% isopropanol) for 15 min in 37 °C. Lipid droplets were stained in red and subsequently observed with a light microscope. For intracellular lipid quantification, stained ORO was eluted with isopropanol and the optical absorbance of the eluates were measured at 520 nm using a FlexStation 3 microplate reader. Determination of hepatic TG contents was conducted as previously described [44].

### 4.9. Liver Histology Examination

To visualize steatosis, inflammation, and ballooning of the liver, paraffin-embedded sections (5 µm) were prepared and stained with H&E (MXB biotechnology, Fujian, China). Images were captured using a light microscope.

### 4.10. Statistical Analysis

Results were expressed as the mean ± standard error (SE) of at least 3 independent experiments. The significance of differences among groups was assessed by one-way ANOVA analysis followed by Dunnett’s test or Student’s *t*-test. Statistical significance was defined as *p* < 0.05.

## 5. Conclusions

In conclusion, our study demonstrates that Rb2 has a significant improving effect on NAFLD and glucose intolerance in db/db mice, and the underlying molecular mechanism of alleviating hepatic lipid accumulation is associated with the restoration of hepatic autophagy via sirt1 induction and AMPK activation. Our findings reveal a novel molecular mechanism for improving the NAFLD action of Panax ginseng, and highlight the potential beneficial effects of Rb2 in the treatment of NAFLD and T2DM. These findings provide further support that the pharmacological promotion of autophagy is a potential strategy for NAFLD treatment.

## Figures and Tables

**Table 1:** Fat weight and serum biochemical values.

Parameter	Wild Type	db/db	Rb2
Epididymal fat weight (g)	0.18 ± 0.05 ***	2.43 ± 0.31	2.50 ± 0.27
Perirenal fat weight (g)	0.1 ± 0.05 **	1.32 ± 0.21	1.40 ± 0.14
Serum NEFA (mM)	0.23 ± 0.06 *	0.68 ± 0.14	0.57 ± 0.08
Serum triacylglycerol (mM)	0.98 ± 0.29 **	1.50 ± 0.33	1.16 ± 0.12 *
Serum cholesterol (mM)	1.97 ± 0.53 **	2.77 ± 0.14	2.24 ± 0.17 *
Serum glucose (mM)	6.08 ± 0.90 **	19.27 ± 2.56	15.15 ± 4.46 *
Serum insulin (ng/ml)	2.6 ± 0.51 ***	20.58 ± 2.51	17.54 ± 1.83

NEFA: Nonesterified fatty acid, Wild type: Wild type mice as normal control, db/db: Vehicle-treated db/db mice, Rb2: Rb2-treated db/db mice. Data are expressed as mean ± standard error from 10 animals per group. * *p* < 0.05, ** *p* < 0.01, and *** *p* < 0.001 compared with db/db group.
