# Correction: Huang et al. Ginsenoside Rb2 Alleviates Hepatic Lipid Accumulation by Restoring Autophagy via Induction of Sirt1 and Activation of AMPK. *Int. J. Mol. Sci.* 2017, *18*, 1063

**DOI:** 10.3390/ijms27219583

**Published:** 2026-10-27

**Authors:** Qi Huang, Ting Wang, Liu Yang, He-Yao Wang

**Affiliations:** 1 State Key Laboratory of Drug Research, Shanghai Institute of Materia Medica, Chinese Academy of Sciences, Shanghai 201203, China; huangqi@simm.ac.cn (Q.H.); wangting@simm.ac.cn (T.W.); yangliu_0611@163.com (L.Y.); 2 University of Chinese Academy of Sciences, Beijing 100049, China

In the original publication [1], there were errors in Figures 5 and 7. During final figure preparation, the Actin panel in Figure 5A was inadvertently duplicated from the Actin panel in Figure 5C, and the LC3 panel in Figure 7B was inadvertently duplicated from the LC3 panel in Figure 8D. The corrected Figure 5 and Figure 7 are provided below.

The authors state that the scientific conclusions are unaffected. This correction was approved by the Academic Editor. The original publication has also been updated.

## Figures and Tables

**Figure 5:**
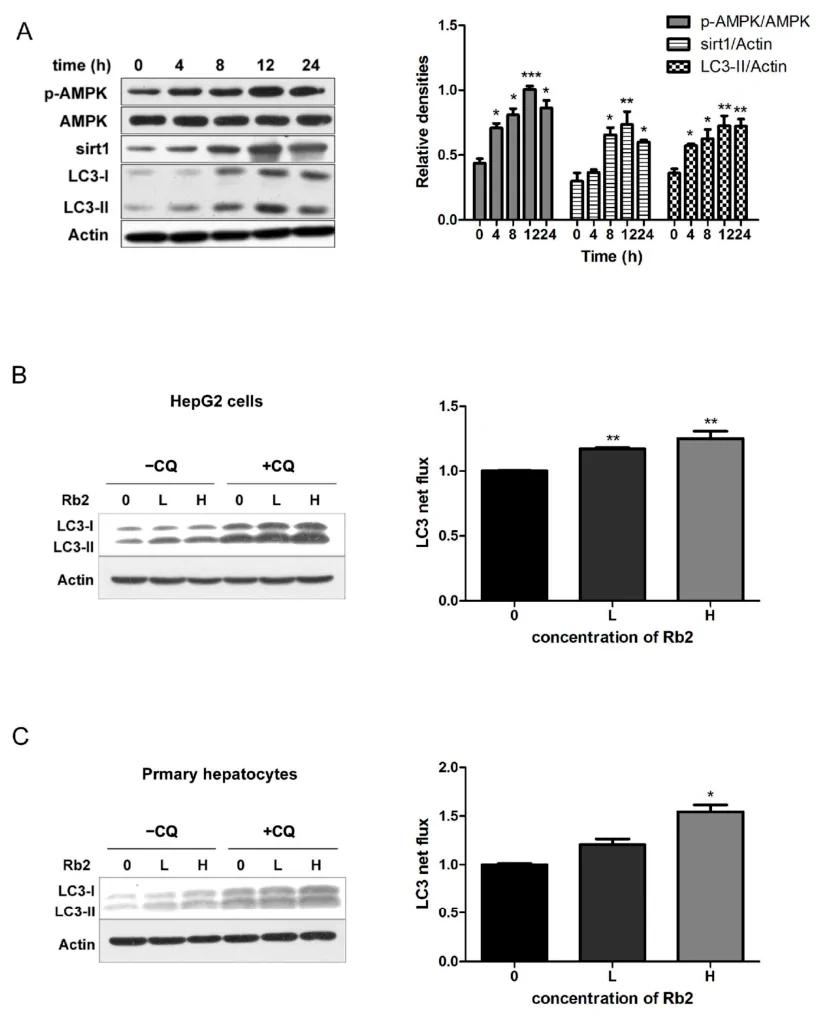
Rb2 promoted autophagic flux by increasing sirt1 expression and AMPK phosphorylation in a time- and dose-dependent manner in hepatocytes. The levels of LC3-II, P62, sirt1, and the p-AMPK/AMPK ratio were evaluated by Western blotting analysis after Rb2 (50 µmol/L) treatment for 0, 4, 8, 12, and 24 h in HepG2 cells (**A**). Autophagic flux was evaluated by Western blotting analysis after Rb2 (0: DMSO, L: 10 µmol/L, H: 50 µmol/L) treatment with and without chloroquine (CQ) (25 µmol/L) for 12 h in HepG2 cells (**B**) and primary mouse hepatocytes (**C**). Data are expressed as mean ± SE from three independent experiments. * *p* < 0.05, ** *p* < 0.01, and *** *p* < 0.001 compared with vehicle (DMSO)-treated group (Control).

**Figure 7:**
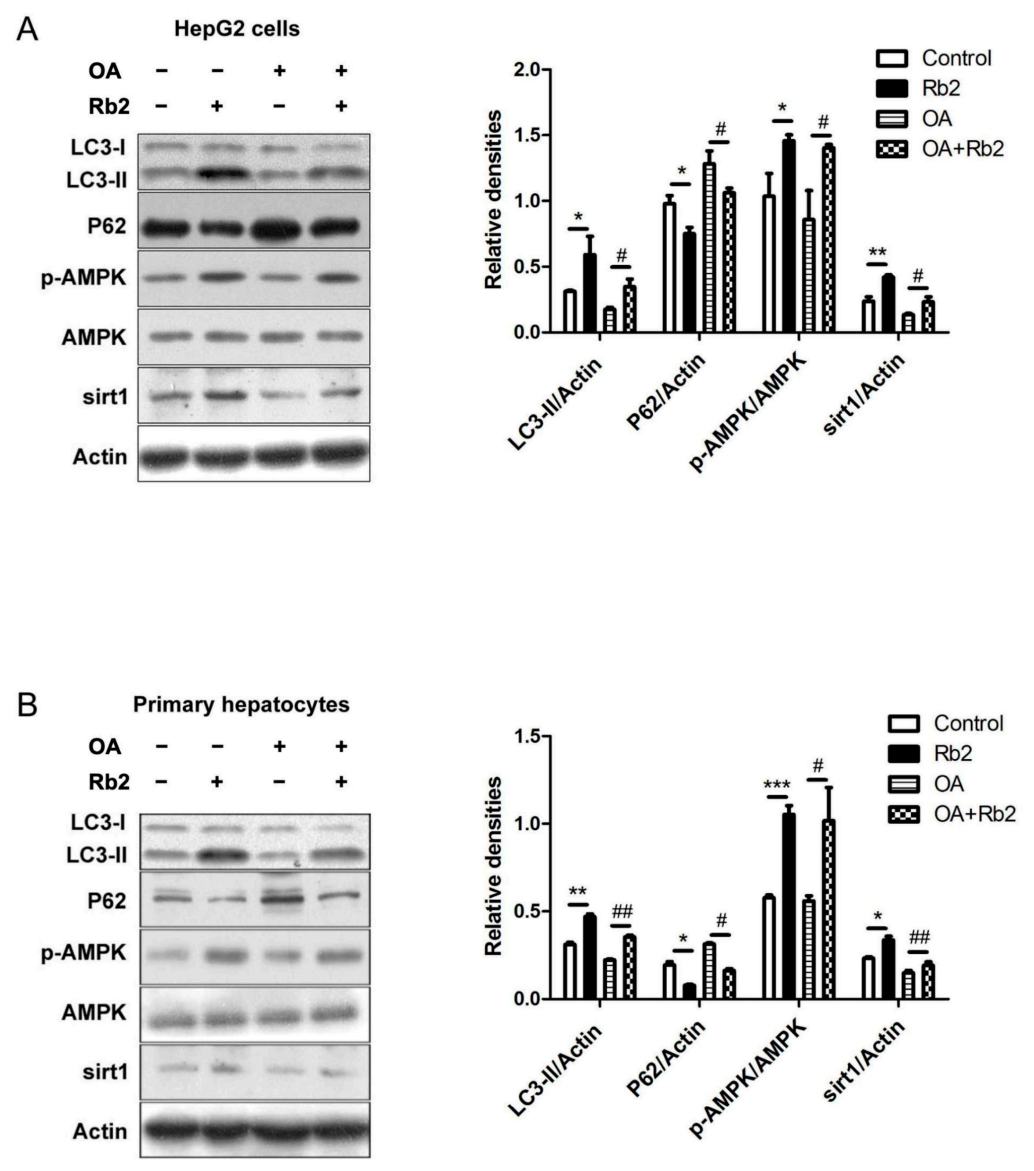
The effect of Rb2 on OA-induced hepatic autophagy dysfunction through the upregulation of sirt1 expression and AMPK phosphorylation. HepG2 cells (**A**) and primary mouse hepatocytes (**B**) were pretreated with 50 µmol/L Rb2 for 4 h before OA (33.3 mmol/L glucose in combination with 1 mmol/L OA for HepG2 and 2 mmol/L OA for primary hepatocytes) exposure for 12 h, and levels of LC3-II, P62, sirt1, p-AMPK, and AMPK were detected by Western blotting analysis. Data are expressed as mean ± SE from three independent experiments. * *p* < 0.05, ** *p* < 0.01, and *** *p* < 0.001 compared with vehicle-treated group (Control). ^#^ *p* < 0.05 and ^##^ *p* < 0.01 compared with OA-treated group (Control).
